# Negative social interactions and coping behaviors: experiences of Japanese mothers caring for children with special needs in disaster areas

**DOI:** 10.1186/s13104-020-05087-1

**Published:** 2020-05-20

**Authors:** Miyako Kimura

**Affiliations:** Department of Preventive Medicine, St. Marianna University School of Medicine, 2-16-1, Sugao, Miyamae-ku, Kawasaki-shi, Kanagawa 216-8511 Japan

**Keywords:** Children with special needs/disabilities, Content analysis, Coping, Disaster, Negative social interactions, Negative support, Mothers, Great East Japan Earthquake, Kumamoto Earthquake, Qualitative study

## Abstract

**Objective:**

This study aims to identify the challenging experiences pertaining negative social interactions and the coping behaviors of mothers of children with special needs after two major earthquakes in Japan. A qualitative content analysis was conducted based on the interviews of 26 mothers of children with special needs who had experienced the Great East Japan Earthquake in 2011 or Kumamoto Earthquake in 2016.

**Results:**

The themes extracted were “perceiving pressures and unfairness,” “failing to obtain support and deeper understanding,” “realizing child’s characteristics that are difficult for others to understand,” and “tackling challenges on their own in different ways.” The experienced negative social interactions and coping behaviors were found to be similar in both earthquakes. Although the Japanese legislation was amended 2 years after the Great East Japan Earthquake, it may not have had necessarily improved the mothers’ situations. Thus, while it is important to provide specific support for families of children with special needs after natural disasters and organize food supplies with a focus on family units, it is also important to increase Japanese society’s understanding of the varied characteristics of disabilities.

## Introduction

Disasters substantially affect vulnerable populations such as children with special needs and their families. Generally, with assigned caregiving roles and responsibilities, an increase in women’s pre-disaster vulnerability is possible; this may make recovery more difficult [1–3]. Therefore, mothers of children with special needs (CWSN) are considered a vulnerable population.

Women are more likely to experience negative aspects of social support than men, which may increase their risk to posttraumatic stress [4]. Such negative social support is included within the broader concept of negative social interactions (NSIs); for example, discouraging individuals’ emotional expressions, criticizing, invading privacy, not providing promised help, and so on [5]. Although NSIs might more strongly affect individuals’ psychological well-being than positive interactions [6, 7], little information is available on NSIs experienced by vulnerable populations after disasters.

Between 2004–2013, Japan experienced 18.5% of all the global earthquakes with magnitudes of 6.0 or greater [8]. After the 2011 Great East Japan Earthquake, many parents of children with developmental disabilities (DD) experienced a scarcity of information and appropriate accommodations, and their children faced a general lack of understanding and support [9]. Similarly, after the 2016 Kumamoto Earthquake, mothers of children with DD reported discomfort and fatigue while supporting their children [10]. Although these reports highlight some of the difficulties faced by and the specific needs of mothers of CWSN, the experiences of NSIs and their coping remain unclear.

This preliminary study addresses the following research questions: (1) What NSIs are experienced by mothers of CWSN after disasters? and (2) how do the mothers cope with these NSIs? For this purpose, I identify the NSIs of mothers of CWSN after two earthquakes and examine their coping behaviors.

## Main text

### Method

I used purposive sampling [11], asking for the cooperation of parents’ associations, which announced this study to their members in three prefectures (Miyagi, Iwate, and Kumamoto, the most affected areas by the two earthquakes). Since 96.8% of children with intellectual disabilities under 18 years and 98.9% of children with mental disorders under 20 years were cared for at home in 2016 [12], mothers of children under the age of 20 might be burdened during emergencies. Thus, the eligible participants of this study were mothers of children with intellectual/developmental disabilities who were under 20 years during the Great East Japan Earthquake or Kumamoto Earthquake. The leaders of parents’ associations explained this study to their members by email, telephone, or word of mouth; these members then shared the information with their networks. Mothers who were interested in the research participated in the group interview; thus, the actual number of participants who refused to participate in this study was unknown. I followed Elo and colleagues’ checklist for researchers attempting to improve the trustworthiness of a content analysis study [11] and performed semi-structured interviews till data saturation.

Altogether, 26 mothers of CWSN participated in this study through two telephone interviews (conducted in November 2017; 30 min for each interview), and six focus group interviews in their living areas (conducted between March 2018 and September 2019, 60–180 min for each interview). The primary questions asked were: “What challenges did you experience after the earthquake, especially in interacting with others?” and “How did you handle such difficulties?”

Drawing from previous studies [11, 13], a qualitative content analysis was conducted. The transcripts were read several times and divided into smaller meaning units, which were condensed and assigned labels as codes. Similar codes were grouped into categories, which were finally unified into themes.

The author fully conducted the study, followed by asking a researcher familiar with qualitative study and CWSN and some participants to evaluate the interpretations of the data (expert and member checking). Collecting data regarding different disasters from different locations (northeastern and southern Japan) and using secondary data such as field notes facilitated data triangulation. To estimate the data’s trustworthiness, face validity was considered [11]. The author also sought opinions of the results from the staff of a non-profit organization and a center for children/individuals, all of whom had supported disaster victims with disabilities.

### Results

The extracted themes for this study, which included 14 categories, are shown in Table 1.

**Table 1:** Themes, categories, and examples of descriptions

Examples of descriptions	Category	Theme
“I spent a month in a school classroom and two months in the gym, where everyone could see us at any time (in the evacuation shelter). That caused [us] substantial stress.” “My child groans while sleeping at night. But everyone lied so close to each other so someone murmured that was annoying (in shelter).”	Others’ gaze and irritation	Perceiving pressures and unfairness
“My house was damaged and in a complete mess. But we didn’t go to the shelter because it was obvious that others would be bothered by my child.”	Fear of trouble in shelter	
“I nearly lost my life, and my car was taken by the tsunami, but because my house remained safe, we were not considered disaster victims. There were no stores and food, but we were not provided with relief supplies.” “We stayed in our house because we couldn’t stay in the site but the other evacuees said it was unfair to claim the supply staying at our house.”	Not regarded as disaster victims	
“It was easy to obtain help for the elderly and individuals/children with physical disabilities but not for children with invisible disabilities.” “Workers of the government prioritized the supply of gasoline to people with physical disabilities or dialysis patients but they didn’t to those with epilepsy.”	Unfair treatment of invisible disabilities	
“We had to prepare meals that took two hours to cook in the morning, one hour for doing the dishes. I didn’t have time to rest before starting the dinner preparations at around 2 p.m. I was exhausted all day when it was my turn…I knew it was for ourselves, but we had to cook for 100–200 people.” “When emergency food assistance was provided to the community, women had to prepare uncooked food, its very time consuming, and too tired.” “When the earthquake occurred, I was pregnant, and searched for foods and drinks for my family, including my elder child with disabilities, all day long. My baby bump was growing, but my husband did not do anything and watched TV.”	Women’s expected roles	
“The local government knew my child requires special aids, but it didn’t help at all.” “I realized the risk of being forgotten in the area.”	Being forgotten	Failing to obtain support and deeper understanding
“I heard officials of the local government didn’t understand not taking the medicine is lethal to people with epilepsy.” “When the earthquake hit us, I was taking care of my bedridden elder son, and my autistic younger son couldn’t stay still. My husband came home right after the earthquake to confirm our safety, but we lost touch with him after he went back to his office. Then, at around 9 p.m., the waves rushed into our house and my elder son died there. Until then, he was breathing…He was breathing…He was breathing…. But there was water up to his waist. My younger son and I could do nothing but watch him gradually die in front of us…Next morning, I escaped from our house with my younger son on my back, wading through chest-deep water, and left my elder son’s body. Everybody around me has avoided this topic as a taboo, and treated me with great caution. But, I’ve always wanted to talk about my elder son…I’m at my limit.”	Lack of deeper understanding	
“You may not know but many of those children are selective eaters. Many children eat nothing but meat.” “I struggled to get juice for a person with developmental disability, who can’t drink water but sweet juice. I didn’t have any stock at home so had to search for it. I realized such preferences might threaten my child’s life.”	Child’s selective eating	Realizing child’s characteristics that are difficult for others to understand
“My child ate or drank up everything even though I told him the amount he could eat at one time because he didn’t understand that.” “My son (autism spectrum disorder) released the water in the bathtub, which I managed to gain by having queued for water many times. He couldn’t stand bathtub filled with water.” “When supply foods were provided to disaster victims, we had to stand in line for a long time… It was difficult for me to manage my son’s behavior and had to give up on obtaining supplies.” “After the earthquake, my child’s problematic behaviors worsened…but I didn’t know how to treat my child…I was worried about my child, but had no idea.”	Child’s uncontrollable behaviors	
“Two or three days after the earthquake, water was supplied to the elderly but it was just a sip per person, only one centimeter of a plastic bottle. So I was feeling ashamed when asked them to give that water to my son with disability. I said I don’t need it for myself but my son.” “I insisted the necessity of medicine for the child with disability to the local government official.”	Asking for help	Tackling challenges on their own in different ways
“It’s difficult to say, please give us special treatment.” “I couldn’t get special treatment because of child with a disability.” “In the evacuation site where we stayed, there was a room for people with disabilities or those who were sick, which had a heater. But my child with developmental disability was not physically disabled and did not look he has a disability, he could walk. So we didn’t go to that room.”	Not asking for help	
“We are trying to help ourselves out on our own because the local government can’t.” “I was so amazing because the PCs of local government were washed away by waves. The information about my son on their PC disappeared…we shouldn’t rely on the local government too much.”	Not rely too much on official support	
“(After the earthquake) I kept requesting the school for children with disabilities to offer the place for an evacuation site. So when I was the leader of PTA, I conducted disaster drills with communities and schools.” “We (members of parents’ associations) requested the local government to improve our situations, and some of our ideas related to disaster preparedness were reflected in local government policy.”	Working with others	
“I wrote up child’s information including medicine and allergy on a paper and kept it in the bottle that I put in refrigerator. It may be useful for someone who helps my child.” “My child and I have whistles to ask for help, everywhere.” “Even today, I have retained the habit of keeping water in the bathtub.”	Preparing for future disasters	

*PTA* parent–teacher association

#### Perceiving pressures and unfairness

Mothers who lost their cars and houses had stayed in evacuation shelters. These mothers described their challenges related to *others’ gaze and irritation* in shelter. For example, a mother explained her situation: “*I spent a month in a school classroom and two months in the gym, where everyone could see us at any time. That caused [us] substantial stress” (Tohoku: T).*

In contrast, mothers whose houses or cars were still usable decided to not stay in shelters, and one mother explained her *fear of trouble in shelter* as follows: “*My house was damaged and in a complete mess. But we didn’t go to the shelter because it was obvious that others would be bothered by my child” (Kumamoto: K).* Most mothers told similar stories, and consequently, they were *not regarded as disaster victims*, and thus, unable to obtain relief supplies. *“I nearly lost my life, and my car was taken by the tsunami, but because my house remained safe, we were not considered disaster victims. There were no stores and food, but we were not provided with relief supplies” (T)*.

Most mothers of children with autism spectrum disorders and/or intellectual disabilities expressed *unfair treatment of invisible disabilities*. *“It was easy to obtain help for the elderly and individuals/children with physical disabilities but not for children with invisible disabilities” (T).*

In addition, *women’s expected roles* expanded under unusual circumstances. For example, in some evacuation shelters, women had to prepare meals for all evacuees.

*We had to prepare meals that took two hours to cook in the morning, one hour for doing the dishes. I didn’t have time to rest before starting the dinner preparations at around 2 p.m. I was exhausted all day when it was my turn. … I knew it was for ourselves, but we had to cook for 100*–*200 people (T).*

Furthermore, the local culture holds men to be superior to women. Owing to her husband’s patriarchal attitude, one mother got divorced after the earthquake. *“When the earthquake occurred, I was pregnant, and searched for foods and drinks for my family, including my elder child with disabilities, all day long. My baby bump was growing, but my husband did not do anything and watched TV (T).”*

#### Failing to obtain support and deeper understanding

Most mothers realized the risk of *being forgotten* during and after emergencies: *“The local government knew my child requires special aids, but it didn’t help at all.”(K).* Moreover, there was a *lack of deeper understanding* of CWSN and their mothers’ true feelings. Another mother described how others’ considerations restrained her emotions:

*“When the earthquake hit us, I was taking care of my bedridden elder son, and my autistic younger son couldn’t stay still.* <*snip*> *Until then (the waves rushed into the house), he was breathing…He was breathing…He was breathing… But there was water up to his waist. My younger son and I could do nothing but watch him gradually die in front of us…*< *snip*> *Everybody around me has avoided this topic as a taboo, and treated me with great caution. But, I’ve always wanted to talk about my elder son…I’m at my limit.”(T).*

Only this mother lost her son, but the other mothers who lived near the seacoast expressed similar fears. Although the damages of earthquakes were different by regions, most mothers in this study wanted to tell their stories.

#### Realizing children’s characteristics that are difficult for others to understand

Although mothers succeeded in obtaining supplies, many of their children were *selective eating* with special preferences. One mother said, *“I realized such preferences might threaten my child’s life” (T).* Moreover, some mothers experienced substantial stress because of their child’s *uncontrollable behaviors*, which included the tendencies to use-up food and drink supplies that were limited, or wasting water. These challenges of children also contributed toward mothers’ feelings of pressures, unfairness, and lack of deeper understanding.

#### Tackling challenges on their own in different ways

Considering the above challenges, mothers in both Tohoku and Kumamoto have taken actions. For example, some mothers tried disclosing their child’s disability to the evacuees and *asking for help*. As most mothers failed to obtain adequate support, they made considerations to *not rely too much on official support*. On the other hand, most mothers have been *working with others,* and one mother became the leader of parent–teacher associations, and conducted disaster drills with communities and schools. Similarly, some mothers requested the local government to improve their situations and influenced local government policy. Additionally, most mothers have been *preparing for future disasters*, and one mother said, “*I wrote up child’s information including medicine and allergy on a paper and kept it in the bottles that I put in refrigerators*” *(T)*. While some mother had whistles to signal for help, some stored water in bathtubs for later use. However, some mothers hesitated to get special treatment because of their CWSN and did not ask for help. These mothers’ attitudes remain the same today.

### Discussion

This study presented the challenging experiences of 26 mothers of CWSN in two different earthquakes, which may help in understanding what NSIs affect these vulnerable populations.

Although it is difficult for a family with CWSN to live in shelters anywhere in the world [3], typical Japanese evacuation styles often resulted in very less privacy. Moreover, after the Great East Japan Earthquake, the Basic Act on Disaster Management was altered in 2013 to facilitate the provision of relief supplies to victims outside shelters [14]. Nevertheless, the mothers who experienced the 2016 Kumamoto Earthquake reported similar experiences as the Tohoku mothers claiming that relief supplies were lacking. Since the lack of supplies is related to perceiving unfairness and not all mothers could ask for help, there is a need to increase the local communities’ awareness of this amended law, which may help in reducing the occurrences of NSIs.

Women’s expected roles in traditional communities became an additional burden. Hikichi et al. [15] reported similar experiences of public nurses in Tohoku, wherein local male community leaders insisted that women wake up at 5 a.m. and prepare meals. These situations are more burdensome to the mothers of CWSN. To mitigate such burdens, distributing supplies to family units rather than communities may be helpful.

Although there were various challenges, the mothers tried to cope with them in different ways. Dealing with stressful or threatening situations is defined as coping [16], which included problem-focused coping strategies (trying to change the situation) and emotion-focused coping strategies (trying to distract one’s attention from the stressful situation) [17]. In this study, most mothers expressed their disappointment of not receiving adequate support; however, they shifted their thinking and acted toward beneficial outcomes. Although previous studies reported that women are more likely to engage in emotion-focused coping strategies in comparison to men [18, 19], the mothers interviewed for this study actively used problem-focused coping strategies. However, some mothers hesitated to seek help, which may deepen the risks faced by these mothers and their children in emergencies. Thus, those interactions with others that reduce their hesitations should be considered by mothers of CWSN.

## Limitations

As the study could not recruit participants from Fukushima, the true extent of the NSIs of mothers of CWSN may have been underestimated. Since it was necessary to reduce burdens on disaster victims, only a few participants were invited for member checking. Moreover, data analysis could not be performed with other researchers and inter-liner reliability could not be verified. These points reduced the validity of the study. Moreover, non-participants in this study might have had more severe experiences than the actual participants, which may affect the findings. Furthermore, this study only highlighted the experiences of a small number of mothers in limited locations. To examine the relationship between various types of NSIs and coping among CWSN families, further quantitative research should be needed.

## Data Availability

The descriptive data and the analyzing process of this study were included in Table 1. The datasets generated and analyzed during the current study are not publicly available because they contain sensitive information of the participants, which might be identified.

## Abbreviation

MK: Miyako Kimura
